# Activation of the PPARγ Prevents Ferroptosis-Induced Neuronal Loss in Response to Intracerebral Hemorrhage Through Synergistic Actions With the Nrf2

**DOI:** 10.3389/fphar.2022.869300

**Published:** 2022-04-20

**Authors:** Chenyang Duan, Dian Jiao, Hanbin Wang, Qiaoli Wu, Weidong Men, Hua Yan, Chunhui Li

**Affiliations:** ^1^ Affiliated Hospital of Hebei University, Baoding, China; ^2^ Hebei University, Baoding, China; ^3^ Tianjin University, Tianjin, China; ^4^ Tianjin Huanhu Hospital, Tianjin University, Tianjin, China; ^5^ Tianjin Neurosurgical Institute, Tianjin Huanhu Hospital, Tianjin, China

**Keywords:** PPAR γ, intercerebral haemorrhage, Nrf2, ferroptosis, GPx4, primary neuron cell culture, pioglitazone, ML385

## Abstract

Intracerebral hemorrhage (ICH) is a subtype of stroke characterized by high mortality and disability rates. The long-term effects of ICH-induced intracranial hematoma on patients’ neurological function are unclear. Currently, an effective treatment that significantly reduces the rates of death and disability in patients with ICH is not available. Based on accumulating evidence, ferroptosis may be the leading factor contributing to the neurological impairment caused by ICH injury. Peroxisome proliferator-activated receptor γ (PPARγ) is a ligand-activated receptor in the nuclear hormone receptor family that synergistically interacts with the nuclear factor erythrocyte 2-related factor 2 (Nrf2) pathway to promote the expression of related genes and inhibit ferroptosis. Primary rat hippocampal neurons were treated with heme (50 μM) and erastin (50 μM) to induce ferroptosis, followed by the PPARγ agonist pioglitazone (PDZ, 10 μM) to verify the inhibitory effect of PPARγ activation on ferroptosis. ML385 (2 μM), a novel and specific NRF2 inhibitor, was administered to the inhibitor group, followed by an analysis of cellular activity and immunofluorescence staining. *In vivo* Assays, ICH rats injected with autologous striatum were treated with 30 mg/kg/d pioglitazone, and the inhibitor group was injected with ML385 (30 mg/kg). The results showed that PDZ inhibited ferroptosis in neurons by increasing the expression of PPARγ, Nrf2 and Gpx4 *in vitro*, while PDZ reduced ferroptosis in neurons after ICH and promoted the recovery of neural function *in vivo*. Our results suggest that PDZ, a PPARγ agonist, promotes Gpx4 expression through the interaction between PPARγ and the Nrf2 pathway, inhibits ferroptosis of neurons after ICH, and promotes the recovery of neural function.

## Introduction

Hemorrhagic stroke is a hemorrhagic disease caused by rupture of the intracranial vasculature, accounting for approximately 15–20% of stroke cases. (Virani et al., 2020) Compared with ischemic stroke, intracerebral hemorrhage (ICH) has a higher mortality and disability rate. (Chen et al., 2020) ICH’s secondary brain damage caused by ICH may lead to neurological impairments and progressive cognitive dysfunction. (Qureshi et al., 2009) Therefore, in treating ICH, preventing neuron loss and salvaging potentially reversible neuronal function are essential therapeutic goals. (Pinho et al., 2019) After ICH, the rupture of intracranial vessels causes blood to enter the brain parenchyma and form hematomas. Hemoglobin, the most abundant protein in hematomas, exerts neurotoxic effects in many preclinical and clinical studies. Chemokine causes the infiltration of microglia/macrophages into the area around the hematoma. Then, microglia/macrophages metabolize hemoglobin to produce iron or ferrous iron, leading to ROS increase and lipid peroxidation. After iron and ferrous iron are transferred out of microglia, neurons deposit iron in neurons through transferrin (TF). (Aguilar and Freeman, 2010) This process eventually leads to abnormal iron ion metabolism in neurons around the hematoma. The deposited iron and hydrogen peroxide cause the Fenton reaction in the cell to produce a large concentration of highly toxic hydroxyl radicals. These hydroxyl radicals cause the peroxidation of unsaturated fatty acids on the surface of cell membranes, subsequently leading to programmed cell death. (Wan et al., 2019)

Ferroptosis is a regulated form of cell death caused by lipid peroxidation, in which high levels of ROS and ferrous iron cause lipoxygenase to over oxidize the abundant phospholipids on the surface of cell membranes, ultimately leading to programmed cell death. (Jiang et al., 2021) Ferroptosis is also manifested by the accumulation of reactive oxygen species (ROS), lipid peroxidation, depletion of glutathione, and inactivation of the phospholipid peroxidase glutathione peroxidase 4 (GPX4). Ferroptosis may be related to lipid peroxidation, dysfunctional production and removal of ROS, and abnormal iron metabolism. (Stockwell et al., 2017) In recent years, some studies have shown that the loss of GPX4 function, the increase in active free iron levels, and the oxidation of polyunsaturated fatty acids (PUFAs) are signs of ferroptosis or three activation pathways related to ferroptosis. Nuclear factor erythrocyte 2 related factor 2 (Nrf2) is a crucial transcription factor specifically responsible for maintaining cellular metabolism and redox and protein balance during stress and plays an essential role in inhibiting ferroptosis. (Anandhan et al., 2020) The transcription factor Nrf2 and many of its target genes have been shown to play essential roles in ferroptosis. The use of Nrf2 agonists to regulate the antioxidant response element (ARE) and inhibit ferroptosis in cells may play a role in protecting neuronal function in individuals with ICH (Giudice and Montella, 2006).

Peroxisome proliferator-activated receptor γ (PPARγ) is a ligand-induced nuclear receptor. PPARγ is activated and binds to RXR to form a heterodimer and is transported to the nucleus, where it binds the PPAR response element (PPRE) in the target gene. (Cai et al., 2018; Villapol, 2018) Then, the nuclear receptor coactivator and PPAR-RXR act synergistically and complement and stabilize the active transcription complex In the nucleus. (Biscetti et al., 2009) Nrf2 exerts regulatory effects as an antioxidant, reduces ROS production, reduces the cell damage induced by ROS, and reduces inflammation by inhibiting the NF-κB pathway. (Lee, 2017) The role of PPARγ in regulating the antioxidant response and the regulation mediated by Nrf2 exert a synergistic effect. Many pharmacological experiments have shown that most of the individual PPARγ and Nrf2 agonists simultaneously activate both the PPARγ and Nrf2 pathways *in vivo* and *in vitro* models; Pioglitazone was used as a PPARγ agonist in this experiment to fully use the synergistic transcriptional regulation of the PPARγ and Nrf2 pathways and the antioxidant function of the Nrf2 pathway to inhibit ferroptosis in neurons after ICH. We studied the potential function of neuronal ferroptosis in the activation of the PPARγ transcriptional regulatory pathway in central neuronal cells in a rat ICH model to provide new treatments for protecting neurons and restoring neurological functions in clinical ICH medical treatment and supporting evidence for preclinical studies.

## Materials and Methods

### Experimental Animals

Male SD rats (China National Institute for Food and Drug Control) weighing 250–300 g and aged approximately 10 weeks were kept in a constant temperature experimental animal chamber with a temperature of 20–25°C and humidity of 50–60%. There were up to four rats in each cage. The cage bedding was changed twice a week to ensure that the rats’ living environment was dry and clean. The rats were fed nutritional feed and water. Appropriate (12 h of light and 12 h of darkness) circadian light cycles were provided. All animal experiments are approved by the Animal Experiment Ethics Committee of Tianjin Huanhu Hospital and implemented in accordance with NIFDC guidelines.

### Rat ICH Model

An intracranial injection of autologous blood was used to simulate human ICH in rat as we previously described. (Wang et al., 2018) Briefly, as preoperative preparation, Sprague-Dawley rats (SPF grade; 200–220 g) were fasted overnight before ICH was established. Initially, each rat was anesthetized with 2% pentobarbital (0.2 ml/100 g, P3761; Sigma-Aldrich, Shanghai). The rats underwent surgery using an ultraclean table and were fixed in a stereotaxic frame. The scalp was opened to expose the anterior brain region. A dental drill was used to drill a 1-mm-diameter hole in the skull surface. Blood (100 μl) was collected from the rat tail vein and injected into the rat striatum with a microsyringe (stereotaxic coordinates; 2 mm lateral to the midline, 0.2 mm posterior to bregma, and 5.5 mm deep below the skull). First, 60 μl of autogenous blood were injected at a rate of 2 μl/min, and the next 40 μl of blood were injected at 5 μl/min. Finally, the needle was left for 10 min before being removed. As postoperative care, the coronal aponeurosis and scalp tissue were sutured. The rats were returned to their cage and provided adequate water and feed. If the rats’ mobility was greatly impaired, the feed was placed in the cage to ensure that the animals could access the feed.

### Modified Neurological Severity Score (mNSS)

According to the mNSS, neurological functions, including motor and sensory systems, reflexes and balance, were graded on a numeric scale from 0 to 18 (the maximal mNSS was 18, indicating maximum neurological impairment, and the minimal mNSS was 0, indicating the absence of impairment). MNSS scores were tested in each group at days 1, 2, 3, 5, and 7 after ICH by an independent investigator with no knowledge of the experimental design.

### Water Maze

The specific water maze experiment method has been recorded in detail in the previous literature (Qu et al., 2019). Before the experiment, the rats were trained in the water maze experiment for 5 days. The rats were trained to learn to swim and were able to find the hidden platform within 90 s. Then, 7 days after the ICH, the water maze experiment was performed again, and the water maze experiment was recorded by the camera. Video of the labyrinth experiment. Use the video analysis online website https://www.pslkzs.com/nav.php to analyze the path of the rat and evaluate the residence time.

### Corner Experiment

Place the rat in a corner with an angle of 30°, and record that when the rat hits the corner, it turns to the left/right, and each experiment is performed 20 times for each rat. Calculate the proportion of rats turning to the affected side to evaluate the recovery of rat nerve function (Zhuang et al., 2020).

### Brain Water Content (BWC) Measurements

Brain edema in each group of rats was assessed using the wet-dry method as previously described.(Zeng et al., 2020) Briefly, 24 h after ICH induction, the rats were anesthetized with 2% pentobarbital (0.2 ml/100 g) intraperitoneally and sacrificed by cervical dislocation. The two hemispheres of the brain were separated from the midline and then divided into two parts. After being weighed (considered the wet weight) and recorded separately, the brain tissues were dried for 48 h at 100°C and weighed again (considered the dry weight). The following formula was used to assess the brain water content: water content = [(wet weight − dry weight)/wet weight] 100%.

### Primary Nerve Cell Culture

The primary hippocampal neuron culture of rats P14-P18 has been described in detail in the previous literature (Yu et al., 2020). To put it simply, the male and female rats are raised in a cage at a ratio of 2:4, and the vaginal plug is checked daily to determine the date of fertilization. Then, when the female rat is 14–18 days pregnant, the pregnant rat is sacrificed and immersed. Sterilize in 75% alcohol, then take out the fetal mouse, take out the hippocampus tissue of the fetal mouse brain under a dissecting microscope, put it into DMEM medium (11995040, Gibco, Shanghai, China) containing 1% Penicillin-Streptomycin Solution (15140122, Gibco, Shanghai, China) and homogenized (all operations are performed on an ice box). Digested with 0.05% Pancreatin at 37°C for 10 min, filtered through a 75-micron sieve, and then centrifuged three times (800, 1000 and 1200 rpm) for 5 min each to remove cell debris and impurities. According to the cell density of 30 w/well, the plate is generally plated in a 24-well plate that has been pre-coated with polylysine. After 8 h, the mixed culture medium of Neurobasal (12348017, Gibco, Shanghai, China) + 0.5% PS + 2% B27 (12587001, Gibco, Shanghai, China) + 0.2% L-Glu (A2916801, Gibco, Shanghai, China) was used to culture until the fourth day to start the experiment.

### Cell Counting Kit-8

After the primary hippocampal neurons were cultured to the seventh day, the CCK-8 cell viability detection dye was used for cell viability determination to evaluate the inhibitory effect of pioglitazone on neuronal ferroptosis. The specific determination method is consistent with the CCK-8 test kit (CA1210, Solarbio, Beijing, China) manufacturer’s instructions.

### Calcein-AM/PI Live/Dead Cell Kit

After the primary hippocampal neurons were cultured to the seventh day, the Calcein-AM/PI live cell/dead cell double staining kit (CA1630, Solarbio, Beijing, China) was used to evaluate the inhibitory effect of pioglitazone on neuronal ferroptosis, and the determination was carried out according to the manufacturer’s instructions (Karuppagounder et al., 2018).

### Detection of MDA and Reduced Glutathione

The markers MDA kit (BC0025, Solarbio, Beijing, China), glutathione kit (BC1175, Solarbio, Beijing, China) and 4-Hydroxynonenal (4-HNE) kit (K009925P, Solarbio, Beijing, China), which can indicate the degree of neuronal ferroptosis, were extracted and detected by the kit. The MDA and reduced glutathione contents of the processed brain tissue and primary hippocampal neuron cells passed the detection. The kit was tested according to the manufacturer’s instructions.

### Western Blot (WB) Analysis

A WB analysis was performed as previously described.(Shan et al., 2019) Cerebral tissues and cells were lysed with RIPA lysis buffer. The protein concentration was determined using an enhanced BCA protein assay kit. The total protein (20 μg) from each sample was separated by SDS–PAGE and transferred onto a polyvinylidene difluoride (PVDF) membrane. The nonspecific binding sites on the PVDF membrane were blocked by incubation with 5% nonfat milk in Tris-buffered saline-Tween (TBST) for 1 h. Then, the PVDF membrane was incubated overnight at 4°C with antibodies against PPARγ (1:1,000, #2443; Cell Signalling Technology, MA, United States), Gpx4 (1:1,000, ab262509; abcam, London, United Kingdom), Nrf2 (1:1000, #12721, Cell Signalling Technology, MA, United States) and Tubulin (1: 1000, #2128, Cell Signalling Technology, MA, United States). Then, the membrane was washed with TBST and incubated with horseradish peroxidase-conjugated horse anti-mouse IgG for 1 h at room temperature. Immunoreactivity was visualized using an enhanced chemiluminescence kit. The band intensities were quantified by a densitometric analysis using ImageJ software.

### RT-qPCR

A RT-qPCR analysis was performed as previously described (Zhao et al., 2020). The brains were removed and then perfused with PBS. The 2 mm ipsilateral striatum surrounding the hematoma was removed on ice and washed with cold PBS. Then, the total RNA was extracted using TRIzol (15596018; Gibco, Shanghai, China), and the extracted RNA was reverse transcribed into cDNA by a reverse transcription kit (K1622; Gibco, Shanghai, China). The qPCR was performed on a Roche Lightcycler II using target gene and the reference control gene primers. The primer sequences designed to detect specific genes are listed in the Supplementary Data.

### Immunocytochemistry

We performed immunocytochemical staining according to the methods described in a previous study (Zhao et al., 2020). The rat brain tissue was perfused with PBS and PFA. After anesthesia with phenobarbital the thoracic cavity of the rat was cut open, and a disposable blood needle was inserted into the left ventricle at the apex of the heart, while the right auricle was cut open. After PBS perfusion, 4% PFA was used to continue perfusion and fix the tissue. Then, the rat brain was removed, fixed by soaking in PFA for 24 h, dehydrated by a gradient of 10, 20 and 30% sucrose solutions, and preliminarily cut into 0.5-cm-thick brain slices. After embedding in OCT, the tissue was placed in liquid nitrogen for rapid cooling. Then, frozen slices of brain tissue with a thickness of 10 μm were obtained by frozen sectioning at −20°C. After the frozen brain tissue sections were removed, the excess OCT glue was removed, and the frozen tissues were circled with crayons. Then, the frozen tissues were fixed with 4% PFA, and Tween-20 was added to increase the permeability of the cell membranes. Finally, after sealing with sheep serum, the primary antibodies were diluted in antibody diluents in different proportions [Gpx4 (1:100, ab262509; abcam, London, United Kingdom); PPARγ (1:100, #2443; Cell Signalling Technology, MA, United States) and Nrf2 (1:100, #12721; Cell Signalling Technology, MA, United States)], and the tissues were incubated at 4°C overnight. Then, different diluted fluorescent secondary antibodies were added, and the samples were incubated at 37°C for 40 min. Finally, a sealing tablet containing DAPI was added to seal the slides. The fluorescence images were obtained under a confocal microscope.

### Statistical Analysis

Data are expressed as mean ± SD. Statistical analysis was performed using the ANOVA test followed by the Newman–Keuls multiple-comparison *post hoc* test. *p* < 0.05 was considered statistically significant.

## Result

### *In Vitro*, Heme Causes Ferroptosis in Neurons

**FIGURE 1:**
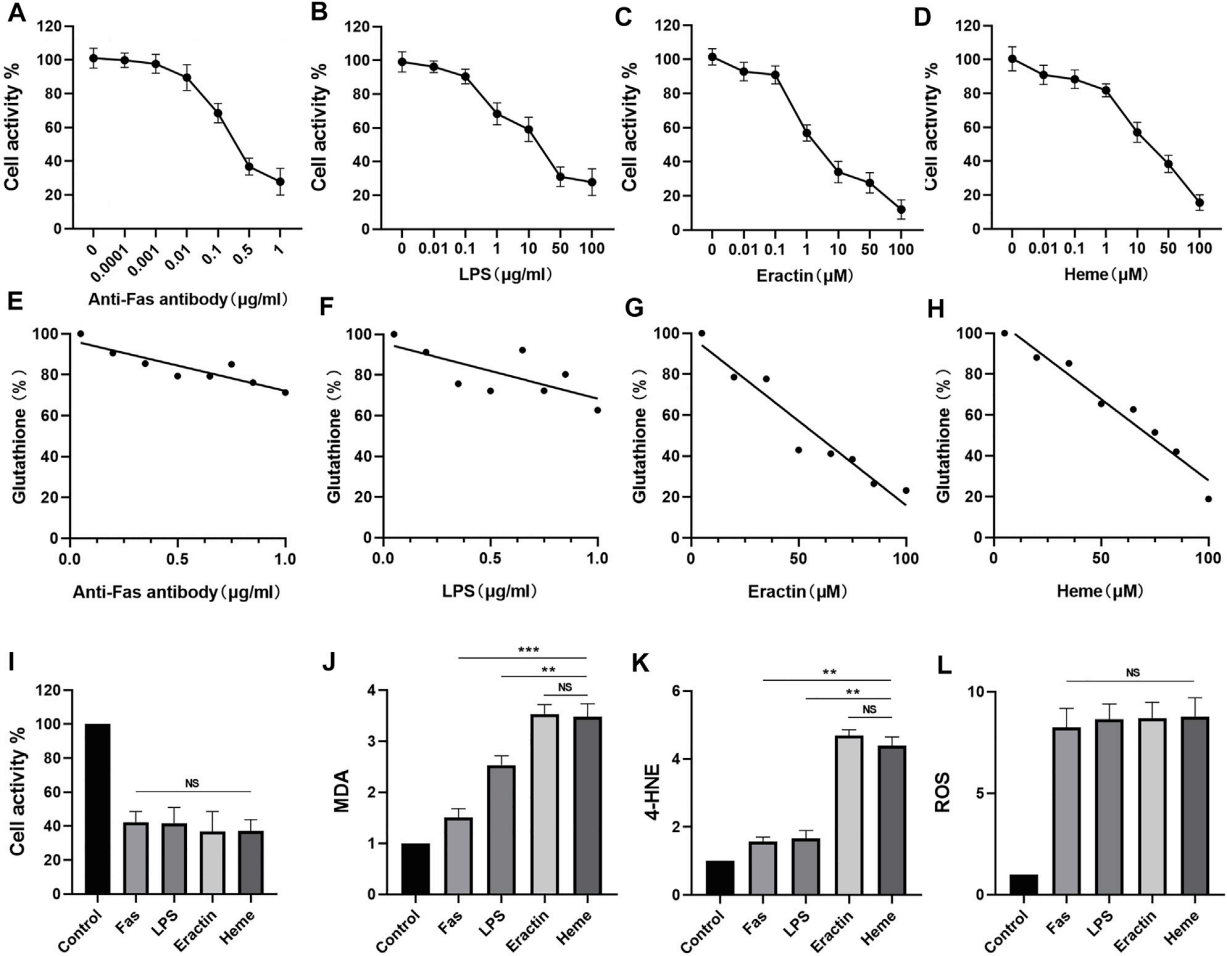
Comparison of the effects of four inducers of neuron death on the expression of barbed wire related metabolites and related proteins. **(A**–**D)** The neural activity of the neurons treated with anti-Fas, LPS, Erastin and Heme at different concentrations for 6 h was determined, and the cellular activity of the neurons was evaluated by CCK-8 analysis. **(E**–**H)** The glutathione (GSH) levels of neurons treated with anti-Fas, LPS, Erastin and Heme at different concentrations for 6 h were determined. **(I**–**L)** Based on cck-8 analysis, neurons were treated with anti-fas antibody (0.5 μg/ml), LPS (50 μg/ml), Erastin (50 μM), and Heme (50 μM) for 6 h, then the levels of MDA, 4-hne, and glutathione were determined. **(M**,**N)** The content of Gpx4 in cell samples of each group was analyzed by WB. Data are shown as the means ± SD of *n* = 3 samples/group, and at least three independent experiments were performed. Statistical analyses were conducted using two-way ANOVA followed by the Newman-Keuls multiple-comparison *post hoc* test. *p < 0.01, **p < 0.01, ***p < 0.001.

In this study, concentrations of anti-Fas Antibody, LPS, Erastin, and Heme induced cell damage were evaluated using CCK-8 cytotoxicity assay (Figures 1A–D). At the same time, changes of intracellular glutathione content were detected by glutathione detection kit when four kinds of inducers induced nerve injury (Figures 1E–G). Subsequently, according to the CCK-8 cytotoxicity - concentration curves of four nerve injury inducers, After 6 h of treatment with anti-fas antibody (0.5 μg/ml), LPS (50 μg/ml), Erastin (50 μM), and heme (50 μM), the levels of MDA, 4-HNE, and glutathione were measured. The results showed that Erastin and Heme significantly increased the contents of MDA (Figure 1I) and 4-HNE (Figure 1J) and significantly depleted glutathione (Figure 1K), while the four inducers caused a similar degree of neuron cell damage. However, the four inducers had similar effects in increasing ROS in neurons (Figure 1L).

### Activation of PPAR can Inhibit the Erastin-Induced Ferroptosis of Neurons

**FIGURE 2:**
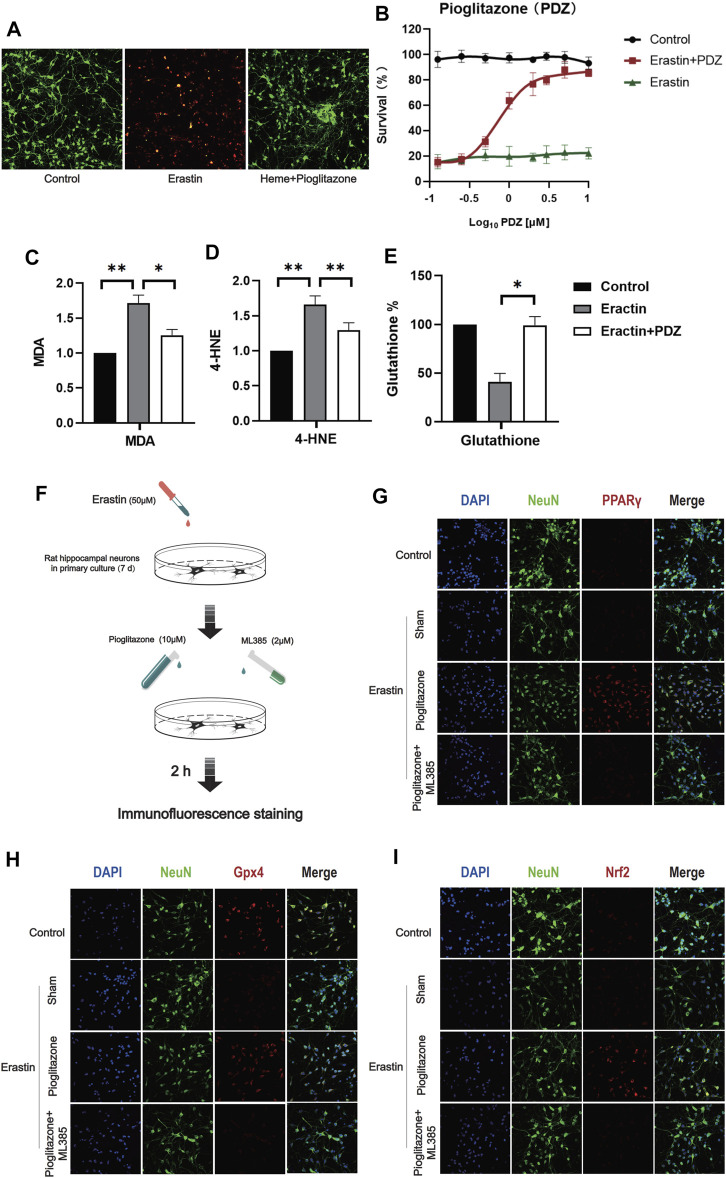
PPARγ activation inhibits Erastin-induced neuronal ferroptosis *in vitro* by activating Nrf2. **(A)** After 7 days of culture, rat primary hippocampal neurons of each group were used for the cell survival/death analysis (green fluorescent cells labeled with calcein-AM were alive, and red fluorescent cells labeled with ethidium homodimer were dead). Then, representative images were captured using a confocal fluorescence microscope. **(B)** The fluorescence intensity of CCK-8 staining was detected at 450 mm using a microplate reader. **(C)** A kit was used to detect the MDA content in each group of neurons. **(D)** 4-Hydroxynonenal (4-HNE) content in each group of cells. **(E)** The reduced glutathione content in the neurons of each group was detected. **(F)** Immunofluorescence staining was used to evaluate the excitatory effect of pioglitazone on PPARγ, Nrf2 and Gpx4, and a flow chart of the cell-based experiment is shown. The rat primary hippocampal neurons were cultured for 7 days and then divided into the following groups: control group, sham group, pioglitazone group, and pioglitazone + ML385 group. **(G)** The characteristic protein NeuN was labeled with FITC, and PPARγ was labeled with Texas Red. **(H)** The characteristic protein of primary hippocampal neurons NeuN was labeled with FITC, and Gpx4 was labeled with Texas Red. **(I)** The characteristic protein NeuN expressed in primary hippocampal neurons was labeled with FITC, and Nrf2 was labeled with Texas Red. Data are shown as the means ± SD of *n* = 3 samples/group, and at least three independent experiments were performed. Statistical analyses were conducted using two-way ANOVA followed by the Newman-Keuls multiple-comparison *post hoc* test. *p < 0.01, **p < 0.01.

In order to verify whether#x3b3; can inhibit the ferroptosis of neurons, Erastin 50 μM was used to induce ferroptosis in primary rat hippocampal neurons 7 days after an extraction. Meanwhile, as PPARγ agonist, different concentrations Pioglitazone (0.1, 0.25, 0.5, 1, 2, 3, 5,10 μM) was used to treat neurons. After 6 h, CCK-8 measured the activity of neuronal cells, and the fluorescence intensity was measured by enzyme labeling instrument in 455 mm. The results showed that pioglitazone could be used as an agonist of PPARγ to inhibit Erastin-induced ferroptosis in neurons (Figure 2B). According to the results of CCK-8, 10 μM pioglitazone had a significant inhibitory effect on the ferroptosis of neurons. 7 days after the primary hippocampal neurons were extracted, three groups of cells were treated, respectively. Control group, Erastin group (treated with Erastin 50 μM, then cultured for 6 h) and Erastin+Pioglitazone group (treated with Erastin 50 and 10 μM Pioglitazone was added then cultured for 6 h). Calcein-AM/PI live/dead cell kit staining was used to observe the activity of neurons. The green fluorescent cells labeled calcein-am were alive, and the red fluorescent cells labeled with ethidium homodimer were dead (Figure 2A).

The results showed that after ferroptosis induced by 50 μM Erastin, 10 μM pioglitazone could significantly improve the survival rate of neurons. At the same time, we also detected the ferroptosis marker MDA and reduced glutathione in three groups of cells. The results showed that pioglitazone could significantly reduce MDA (Figure 2C) and 4-HNE (Figure 2D) and increase the content of reduced glutathione (Figure 2E).

In order to verify the activating effect of pioglitazone on PPARγ in neurons. Control group, Sham group: the neurons were treated with Erastin 50 μM and then cultured for 2 h. Pioglitazone group: treated with Erastin 50 μM and added with Pioglitazone 10 μM, then cultured for 2 h. Pioglitazone+ML385 group: neurons were treated with Erastin 50 μM, Pioglitazone 10 μM, and ML385 2 μM were added simultaneously. The results showed that pioglitazone could also promote the expression of PPARγ, Nrf2, and Gpx4 in the Erastin-induced neuronal ferroptosis model. (Figures 2G–I) ML385, a specific inhibitor of Nrf2, inhibits the expression of PPARγ, Nrf2, and Gpx4 in neurons.

### Activation of PPAR can Inhibit the Heme-Induced Ferroptosis of Neurons

**FIGURE 3:**
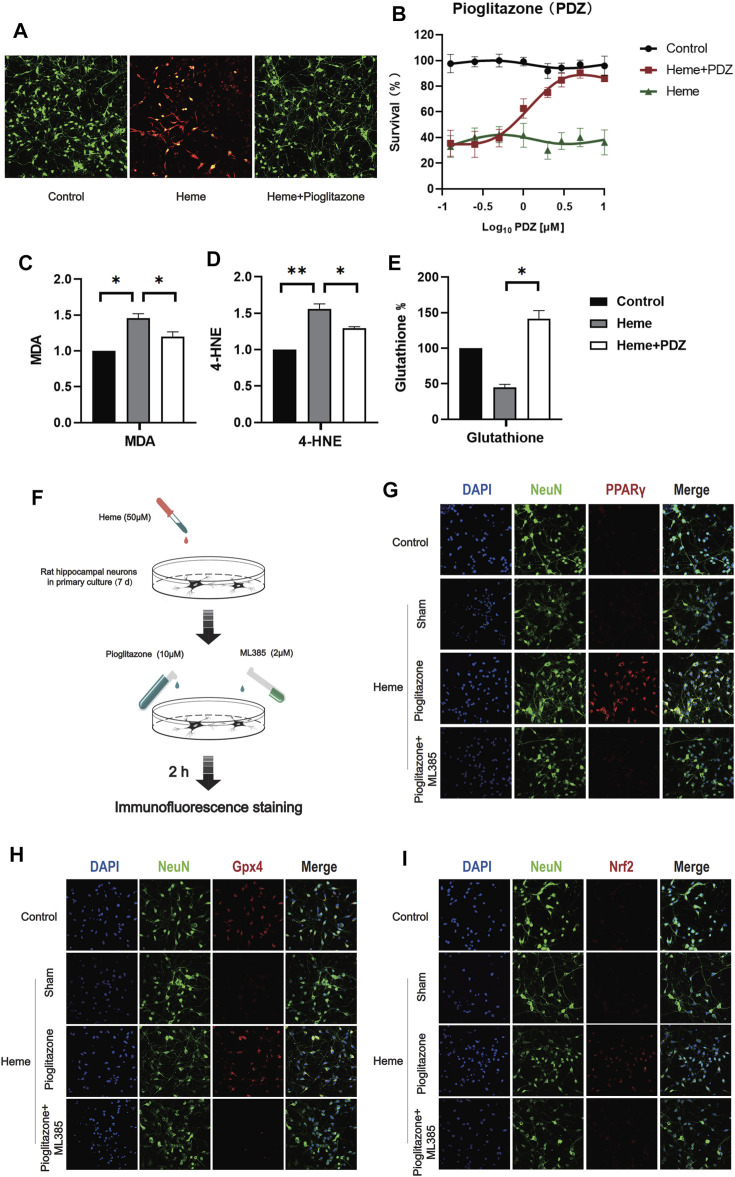
PPARγ activation inhibits heme-induced neuronal ferroptosis *in vitro* by activating Nrf2. **(A)** After 7 days of culture, rat primary hippocampal neurons were used for the cell survival/death analysis (green fluorescent cells labeled with calcein-AM were alive, and red fluorescent cells labeled with ethidium homodimer were dead). Then, representative images were captured using a confocal fluorescence microscope. **(B)** The fluorescence intensity of CCK-8 staining was detected at 450 mm using a microplate reader. **(C)** A kit was used to detect the MDA content in each group of neurons. **(D)** 4-Hydroxynonenal (4-HNE) content in each group of cells. **(E)** The reduced glutathione content in the neurons of each group was detected. **(F)** A flow chart of the cell-based experiment is shown. **(G)** The characteristic protein NeuN was labeled with FITC, and PPARγ was labeled with Texas Red. **(H)** The characteristic protein of primary hippocampal neurons NeuN was labeled with FITC, and Gpx4 was labeled with Texas Red. **(I)** The characteristic protein NeuN expressed in primary hippocampal neurons was labeled with FITC, and Nrf2 was labeled with Texas Red. Data are shown as the means ± SD of *n* = 3 samples/group, and at least three independent experiments were performed. Statistical analyses were conducted using two-way ANOVA followed by the Newman-Keuls multiple-comparison *post hoc* test. *p < 0.01, **p < 0.01.

**FIGURE 4:**
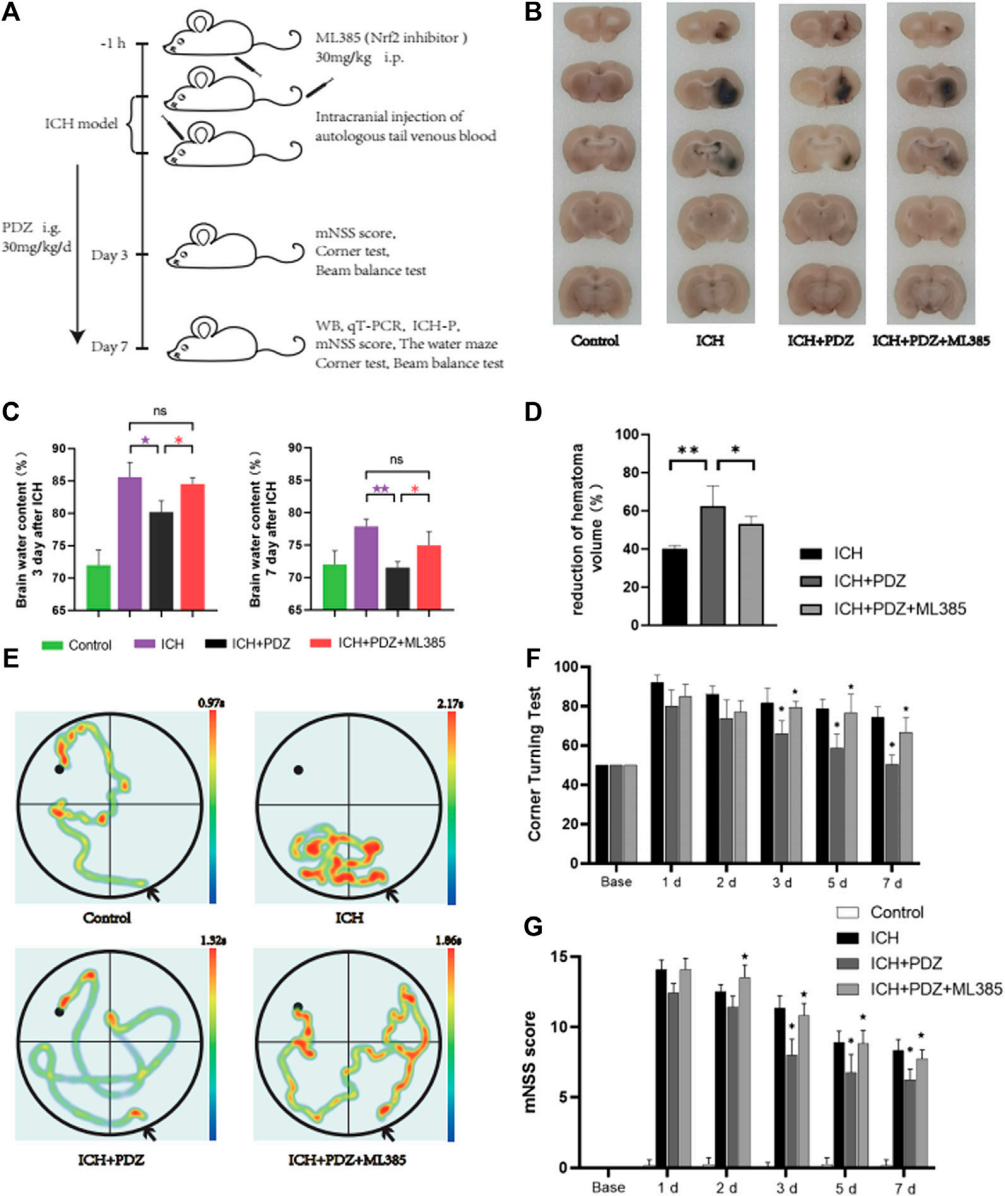
*In vivo* experimental procedures and evaluation of neurological function. **(A)** Schematic of the *in vivo* study. **(B)** Coronal sections of brain tissue were prepared following sucrose dehydration (5 mm), *n* = 3 animals/group. The data were obtained from two independent experiments. **(C)** The cerebral water content was measured 3 and 7 days after ICH. **(D)** The reduction in the hematoma volume is reported as a percentage. **(E)** Representative traces of each group in water maze tests. **(F)** Statistical analysis of the corner turn test. **(G)** mNSS score of rats in each group. PDZ, pioglitazone. Data are shown as the means ± SD of *n* = 3 rats/group, and at least three independent experiments were performed. Statistical analyses were conducted using two-way ANOVA followed by the Newman-Keuls multiple-comparison *post hoc* test. *p < 0.01, **p < 0.01. compared with the ICH group. ★*p* < 0.01 and ★★*p* < 0.01 compared with the ICH + PDZ group.

In order to verify whether PPARγ can inhibit ferroptosis induced by heme, we used Heme 50 μM to induce ferroptosis in rat primary hippocampal neurons 7 days after extraction, and the same method was used to verify the inhibitory effect of pioglitazone on ferroptosis. The results showed that pioglitazone could be used as an agonist of PPARγ to inhibit the ferroptosis of neurons induced by heme. (Figure 3B), at the same time, after 50 μM Heme induced ferroptosis, 10 μM pioglitazone significantly increased the survival rate of neurons. MDA was significantly increased in the heme-induced neuronal ferroptosis cell model, while pioglitazone decreased MDA (Figure 3C) and 4-HNE (Figure 3D) and increased the content of reduced glutathione (Figure 3E).

In Heme-induce (50 μM, 2 h) neuronal ferroptosis, 10 μM pioglitazone significantly increased the expression of PPARγ, Nrf2, and Gpx4 in the neurons (Figures 3G–I).

*In vivo* test, activation of PPARγ can accelerate the clearance of hematoma, reduce brain edema, and promote recovery nerve function.

In order to determine the therapeutic effect of pioglitazone as an agonist of PPARγ on ICH rats, we used the rat model of intracerebral hemorrhage induced by autologous blood injection into the striatum *in vivo*. 24 rats were randomly divided into two groups, and the rats in each group were treated differently. Control group. ICH group: 100 μl of autogenous blood was injected into the striatum of rats. ICH+PDZ group: pioglitazone 30 mg/kg/d intragastric administration after ICH, and the first dose was administered 2 h after ICH 2 h. ICH+PDZ+ML385 group: 30 mg/kg intraperitoneal injection of ML385 1 h before ICH and pioglitazone treatment after ICH. Seven days after ICH, the rats were anesthetized with pentobarbital and killed. The brain was removed for sucrose gradient dehydration, and then the coronal slices of 1–2 mm were cut, and the volume of hematoma was calculated. The results showed that the hematoma volume of ICH+PDZgroup rats was significantly reduced, and the therapeutic effect of PDZ on the ICH rat model was reduced by injection of Nrf2 inhibitor ML385 (Figures 4B,D).

In order to evaluate the degree of brain edema in each group after ICH, the brain water content of each group was calculated at 3 and 7 days after ICH. The specific method has been described in detail in the previous article. The results showed that PDZ could significantly reduce brain water content, while ML385, an inhibitor of Nrf2, weakened the therapeutic effect of PDZ (Figure 4C).

In order to evaluate the recovery of neurological function of rats after ICH, the neurological function of rats in each group was evaluated by mNSS score, corner test, and water maze test 7 days after ICH (Figures 4F,G). In the water maze test, the rats in each group were trained in the water maze 3 days before ICH, and then the recovery of cognitive nerve function was measured by the water maze test on the seventh day after ICH. The results showed that PDZ could significantly promote the recovery of cognitive nerve function in rats after ICH. ML385, an inhibitor of Nrf2, weakens the therapeutic effect of PDZ (Figure 4E). The results of the mNSS score and corner test showed that PDZ could promote the recovery of rats’ motor function and balance function. ML385, an inhibitor of Nrf2, weakens the therapeutic effect of PDZ.

## *In Vivo*, Activation of PPARγ can Inhibit the Ferroptosis of Neurons by Activating Nrf2 and Gpx4

In order to verify the inhibitory effect of PDZ activating PPARγ on ferroptosis in the ICH rat model, a total of 12 rats were randomly divided into four groups: Control group, ICH group, ICH+PDZ group, and ICH+PDZ+ML385 group (the disposal methods of each group have been described in detail in the previous article). WB and PCR analyzed the brain tissue around the hematoma 7 days after ICH. The WB results showed that PDZ could significantly increase the expression of PPARγ, Nrf2, and Gpx4 in the ICH rat model. At the same time, ML385, an inhibitor of Nrf2, not only inhibited the increase of Nrf2 expression by PDZ, but also inhibited the activation of PPARγ by PDZ. The PCR results showed that PDZ activated PPARγ and increased the expression of related cellular pathway genes (including PGC-1 α, CD36, Sirt1, and HIF-1 α) (Fig), and increased the expression of genes related to Nrf2 (including Nrf2, UCP2, HO-1, GST, and FOXO3), which indicated that there was a close synergistic effect between PPARγ and Nrf2 pathway. In addition, PDZ promotes the expression of various ferroptosis suppressor genes (including Gpx4, SLC11A7, GCLC, NCOA4, and SAT1) by increasing the expression Nrf2.

In order to verify the inhibitory effect of activating the PPARγ pathway on the ferroptosis of neurons in the ICH rat model, 12 rats were randomly divided into four groups: Control group, ICH group, ICH+PDZ group, and ICH+PDZ+ML385 group. 7 days after the ICH model, Fluoro-Jade C (the specific test method described in detail in the previous article (Ikenari et al., 2020)) was used to stain frozen sections of rat brain tissue. The results showed that PDZ, as the activation of PPARγ, could inhibit neuronal apoptosis. At the same time, ML385 could counteract the therapeutic effect of PDZ by inhibiting Nrf2.

**FIGURE 5:**
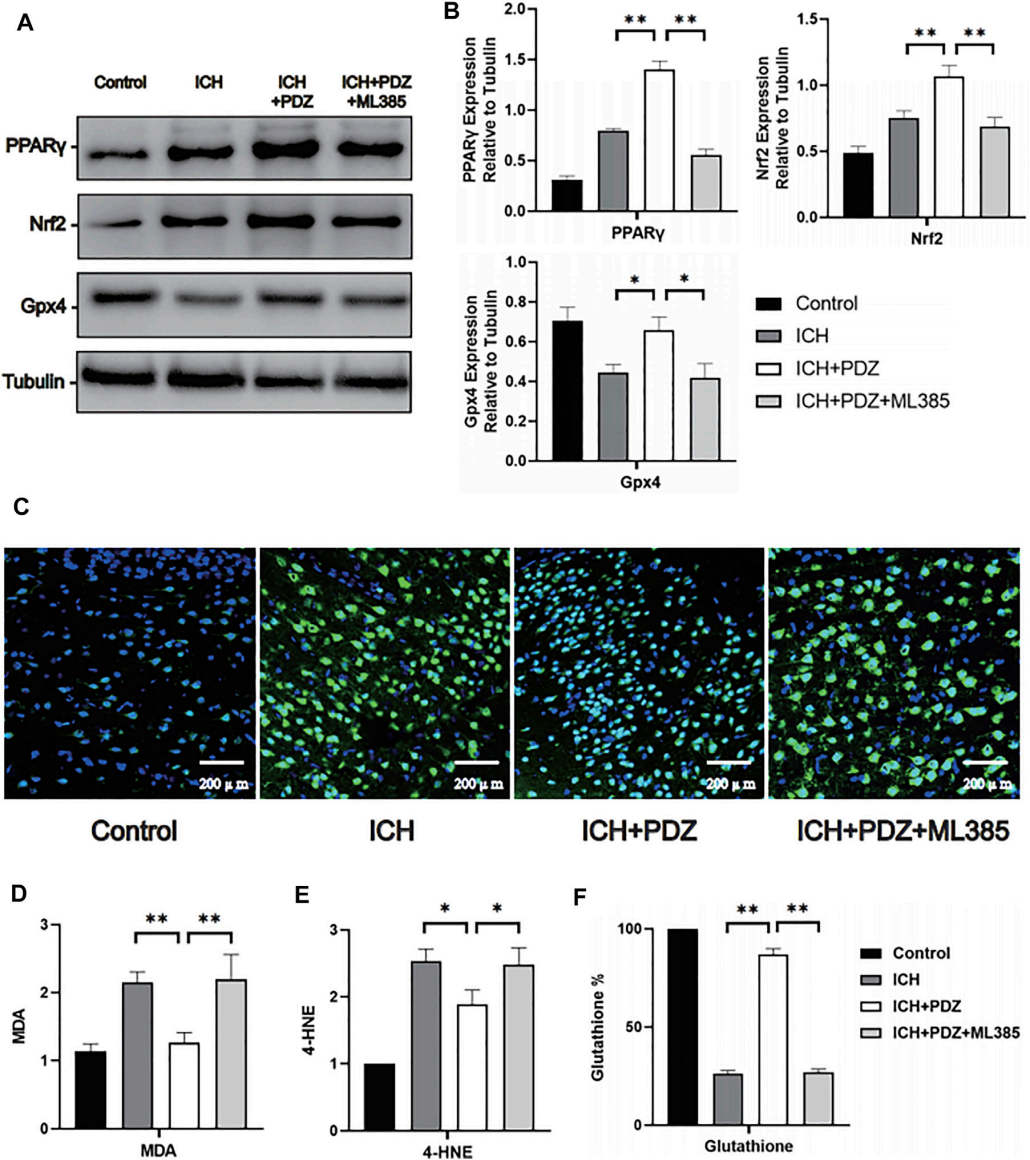
Effects of PPARγ activation on ferroptosis-related cellular pathways *in vivo*. Seven days after ICH, the brain tissue around the hematoma was collected for WB detection, and brain perfusion was performed to prepare frozen sections for immunofluorescence staining. **(A)** Levels of the PPARγ, Nrf2 and Gpx4 proteins. **(B)** ImageJ software was used to further quantify the gray values of Western blot bands. **(C)** Representative images of Fluoro-Jade C staining of the brain tissue around the hematoma. **(D)** The kit was used to detect the MDA content in each group (the specific detection method has been described in detail above). **(E)** The reduced glutathione content in the neurons from each group. Data are shown as the means ± SD of *n* = 3 samples/group, and at least three independent experiments were performed. Statistical analyses were conducted using two-way ANOVA followed by the Newman-Keuls multiple-comparison *post hoc* test. *p < 0.01, **p < 0.01.

In order to further evaluate the inhibitory effect of PDZ on ferroptosis of neurons, the perihematoma tissues of Control group, ICH group,ICH+PDZ group and ICH+PDZ+ML385 group rats were taken 7 days after ICH for quantitative analysis of Malondialdehyde (MDA), 4-Hydroxynonenal (4-HNE) and reduced glutathione (GSH). The results showed that PDZ could reduce MDA and 4-HNE in brain tissue around hematoma and increase reduced glutathione, which indicated that PDZ could inhibit ferroptosis of neurons in ICH model rats (Figures 5D–F). In addition, ML385, an inhibitor of Nrf2, weakens the therapeutic effect of PDZ, suggesting that PDZ, as an agonist of PPARγ, may inhibit ferroptosis by activating Nrf2.

## Discussion

In this experiment, we used the PPARγ agonist pioglitazone as a neuroprotective agent to inhibit ferroptosis in an ICH rat model and used heme and erastin to induce ferroptosis in primary neuronal cells *in vitro*. In the *in vivo* ICH model, pioglitazone induced the expression of PPARγ in brain tissue, similar to the results from previous studies. In a report examining the role of the PPARγ pathway in NF-κB-mediated inflammation in an ICH animal model, pioglitazone significantly increased the expression of PPARγ in brain tissue. (Luo et al., 2021) This experiment proved that PPARγ activation inhibited the loss of neurons in the rat ICH model and played a role in protecting neuronal function. These findings are similar to several previous preclinical studies on stroke. In 2020, a study confirmed that pioglitazone, an agonist of PPARγ, promoted Nrf2 expression and microglia polarization in the cerebral hemorrhage model of a rat. Another 2019 study suggested that a PPARγ activator attenuates cerebral hemorrhage caused by tissue plasminogen activator in a mouse MCAO model and improves neuronal function (Li et al., 2019).

In this experiment, we used anti-Fas Antibody to induce neuronal autophagy, LPS-induced neuronal apoptosis, Erastin induced neuronal, and heme to simulate the neuronal injury model in ICH *in vitro*. By measuring MDA, 4-HNE, glutathione, and Gpx4 are the metabolites and proteins associated with ferroptosis. We found that heme-induced neuronal damage was significantly increased in MDA and 4-HNE, accompanied by depletion of glutathione and Gpx4. These results were very similar to erastin-induced neuronal ferroptosis models proving that ferroptosis is the main form of neuronal death induced by heme. Meanwhile, *in vivo* experiments, MDA and 4-HNE in brain tissues around hematoma were detected to be significantly increased, while glutathione decreased significantly and Gpx4 was depleted significantly in rats 7 days after ICH. These are very similar to changes in metabolites in erastin-induced neuronal ferroptosis, so we demonstrate that heme in hematomas leads to neuronal ferroptosis after ICH in rats, which is the primary cause of secondary nerve injury in ICH.

**FIGURE 6:**
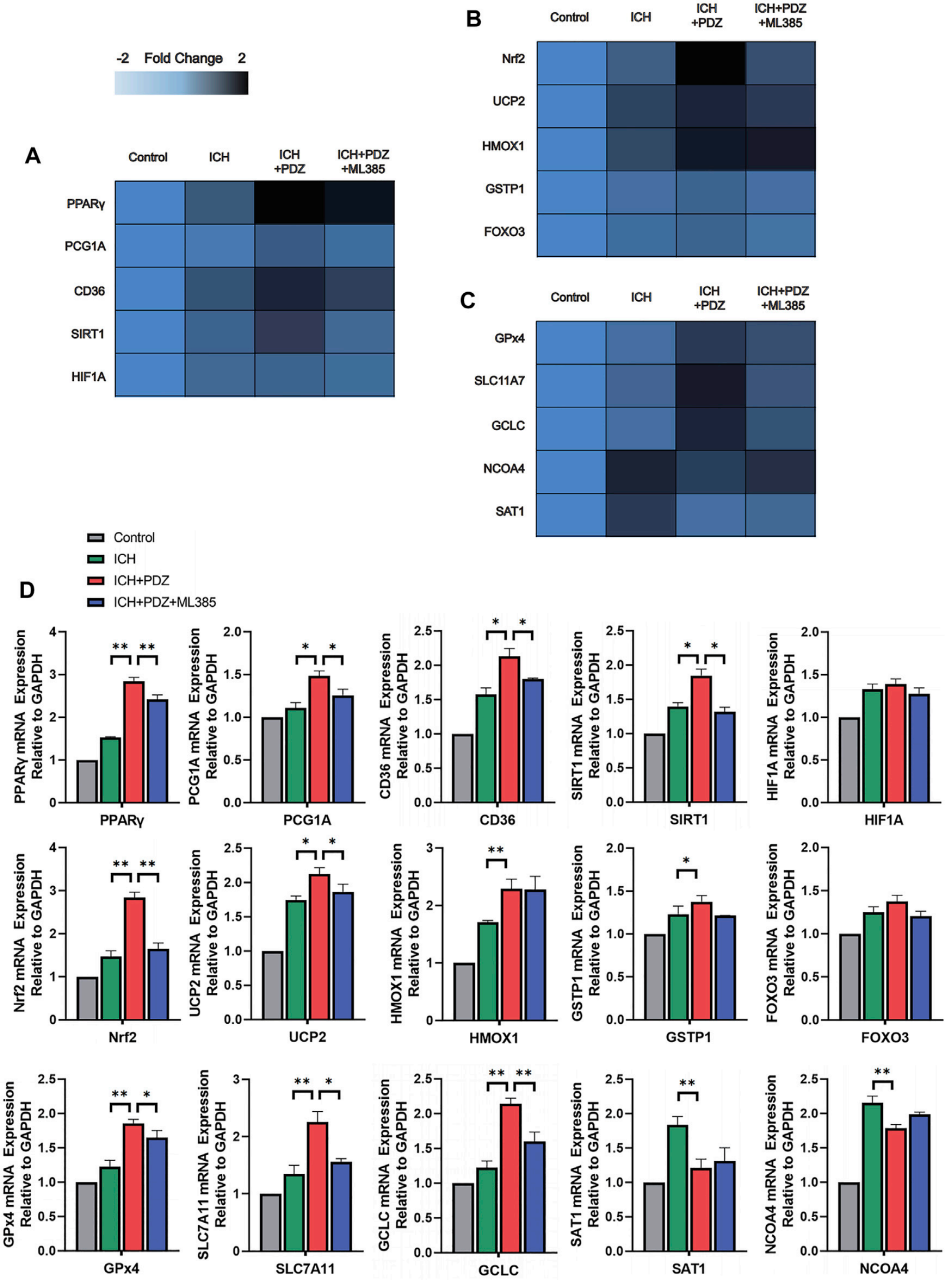
Effects of PPARγ activators on PPARγ-, Nrf2-and Gpx4-related pathways. At 7 days after ICH, the expression of the target genes was detected using RT–qPCR and compared with that of an internal reference gene (GAPDH), and an improved delta-delta-CT algorithm was used. **(A)** Heatmap of the mRNA abundance of genes in the PPARγ signaling pathway. **(B)** Heatmap of the mRNA abundance of genes in the Nrf2 signaling pathway. **(C)** Heatmap of the mRNA abundance of genes in the Gpx4 signaling pathway. **(D)** Detailed comparison of PCR results for each gene. Data are shown as the means ± SD of *n* = 3 samples/group, and at least three independent experiments were performed. Statistical analyses were conducted using two-way ANOVA followed by the Newman-Keuls multiple-comparison post hoc test. *p < 0.01, **p < 0.01.

Cell-based experiments show that PPARγ agonists increase the expression of PPARγ and Nrf2 in neuronal cells and inhibit neuronal death after damage by heme and erastin. The effect of Nrf2 agonists on ferroptosis has been proven in many preclinical studies. (Cui et al., 2021) Under basal conditions, the content of Nrf2 in cells is low. This low level is because Nrf2 binds to a cytoplasmic actin-binding protein, Kelch-like ECH-associated Protein 1 (KEAP1), in the cytoplasm, which is also used as a substrate to participate in the interaction of Keap1-cullin 3-ring box protein 1 (KEAP1-CUL3-RBX1) E3 ubiquitin ligase complex with Nrf2, ultimately causing Nrf2 ubiquitination and degradation by the proteasome. Therefore, the level of oxidative stress-regulated by Nrf2 remains at a low level for a long time under basal conditions. (Anandhan et al., 2020) When cells are exposed to stress, such as reactive oxygen species (ROS) or chemical inducers, Nrf2 dissociates from Keap1, which increases the stability of Nrf2 in the cell and transfers Nrf2 to the nucleus. Then, Nrf2 and small Maf in the nucleus (sMaf) interact and subsequently bind to the antioxidant response element (ARE) to promote the transcription of a series of genes related to the inhibition of polyunsaturated fatty acid oxidation and ferroptosis. (De Nuccio et al., 2020) In mammals, including humans, the Keap1-Cul3-Rbx1 axis is considered the most critical mechanism regulating Nrf2 activity (Giudice and Montella, 2006). In recent years, several studies have proven that the function of Nrf2 in regulating gene expression plays a vital role in iron metabolism. (Kerins and Ooi, 2018) A potential explanation for this finding is that the interaction of Nrf2 with the ARE regulates the expression of genes related to iron metabolism, such as ATP binding cassette (ABC) transporter family B6 and solute carrier family 40 member 1 (SLC40A1) (Hao et al., 2021; Julián-Serrano et al., 2021). In addition, Nrf2 also regulates the expression of genes related to glutathione synthesis/metabolism, such as glutamate-cysteine ligase catalytic subunit (GCLC), solute carrier family seven member 11 (SCL7A11), and GPX4 (Giudice and Montella, 2006). These proteins are inhibited to different degrees in ferroptosis-induce various biochemical and metabolic processes.

**FIGURE 7:**
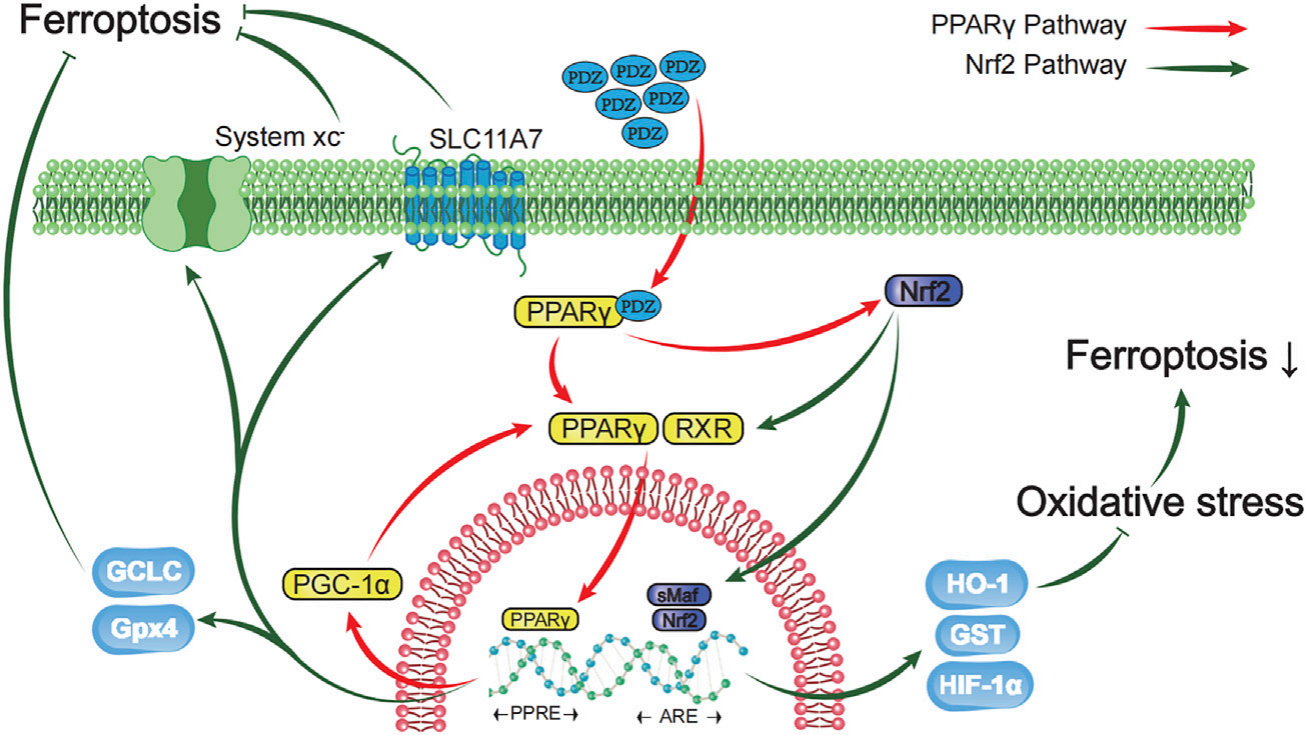
Schematic diagram showing that activation of the PPAR-γ pathway prevents ferroptosis-induced neuronal loss in an intracerebral hemorrhage model through synergistic actions with the Nrf2 pathway. Pioglitazone, a PPARγ agonist, inhibited the iron-induced death of neurons after ICH by activating the PPARγ pathway and promoting the expression of Gpx4, HIF-1α, HO-1, GST and SLC11A7 through the interaction between PPARγ and the Nrf2 pathway. The green arrow represents the Nrf2 pathway, and the red arrow represents the PPARγ pathway.

In this study, PPARγ agonists fully exploited the synergistic effect of PPARγ and Nrf2 on regulating the transcriptional functions to inhibit oxidative stress, which effectively inhibited ferroptosis in primary neuronal cells in an *in vitro* model. In the rat ICH model, these pathways functioned in neuronal ferroptosis. Several studies conducted in recent years have proven that the Nrf2 and PPARγ pathways have close mutual regulatory functions and enhance their expression. PPARγ and Nrf2 seem to have positive feedback in inhibiting oxidative stress. The interaction between Nrf2 and the PPARγ pathway is manifested in several aspects described below (Cai et al., 2018).

1. Activation of PPARγ through the classical pathway promotes the expression of Nrf2 and *vice versa*. For example, the Nrf2-PPARγ pathway regulates lipid metabolism. (Zhong et al., 2021) At the same time, the Nrf2/ARE-PPARγ pathway treats ulcerative colitis. (Lin et al., 2021) The PPARγ-Nrf2 pathway inhibits myocardial ischemia-reperfusion injury (Rani and Arya, 2020).

2. Some genes protecting against oxidative stress contain both ARE and PPRE regulatory sequences, which both Nrf2 and PPARγ can regulate to inhibit oxidative stress. These enzymes include catalase (CAT), glutathione transferase (GST), and superoxide dismutase (SOD) (Jurkunas et al., 2010; Zhang et al., 2013; Zhang et al., 2018).

3. The Nrf2 and PPAR pathways increase the expression of CD36 on the surface of macrophages/microglia and play an essential role in the phagocytosis of lipids by macrophages/microglia (Ishii et al., 2004; Zhou et al., 2008). At the same time, activation of the Nrf2-CD36 axis is essential for removing myelin debris and inhibiting neuroinflammation in demyelinating diseases (such as multiple sclerosis, MS) (Grajchen et al., 2020).

4. Many preclinical and clinical studies have confirmed that the Nrf2 and PPARγ pathways inhibit the inflammatory response by inhibiting NF-κB. For example, glucocorticoids have inhibited inflammatory bowel disease through the PPARγ pathway (Liu et al., 2015). The MAPK-Nrf2 pathway plays a role in treating pneumonia by inhibiting the NF-κB pathway (Xu et al., 2020).

This experiment mainly studied the interaction between the PPARγ and Nrf2 pathways, and the synergistic actions of these two pathways inhibited the neuronal loss caused by ferroptosis in the cerebral hemorrhage model of a rat. We cultured primary rat hippocampal neuronal cells and treated them with heme and erastin *in vitro* to mimic neuronal ferroptosis caused by ICH. Ferroptosis-induced neuronal loss plays an important role in many degenerative neurological diseases, including ICH (such as ischemic stroke, vascular dementia, Alzheimer’s disease, Parkinson’s disease, systemic sclerosis, *etc*.), and experiments are helpful to identify more effective treatments for these diseases. New therapeutic targets provide effective *in vitro* models (Zhao et al., 2007). In this experiment, we used MDA and reduced glutathione to evaluate the degree of neuronal ferroptosis *in vitro* models and the inhibitory effect of PPARγ agonists on ferroptosis. MDA, a product of free radicals that oxidize polyunsaturated fatty acids, has been used to evaluate the degree of lipid oxidation on the cell membrane surface, which is a significant pathway inducing ferroptosis (Li et al., 2021). The content of reduced glutathione has been used to evaluate the level to which oxidative stress is reduced in the cell, especially the degree of inhibition of lipid oxidation in the cell. At the same time, due to the limitation of experimental conditions, total Fe3+ ions were not detected in this experiment. However, several preclinical studies on ICH proved that PPARγ activation did not reduce the total iron content in the hematoma and tissues surrounding the hematoma. X-ray fluorescence microscopy analysis or the Ferene-S kit may effectively detect the total iron content in cells and tissues (Chen et al., 2019). We will gradually and deeply explore the molecular mechanism by which Nrf2 regulates iron metabolism to inhibit ferroptosis in future studies.

Pioglitazone is a synthetic PPARγ agonist and is the first-line drug for the clinical treatment of type 2 diabetes (Zhang et al., 2021). It exerts its pharmacological effects by activating PPARγ in the liver and fat cells. It has produced neuroprotective effects in many preclinical studies and clinical studies. In a clinical cohort study conducted from 2004 to 2010, pioglitazone reduced the risk of vascular dementia in patients with noninsulin-dependent diabetes. However, at the same time, the controversy regarding the side effects of fracture risk and prostate cancer risk caused by PPARγ agonists has persisted in the past 10 years, which has also led to apparent restrictions on the clinical application of PPARγ activators for neurovascular protection in patients with diabetes. However, according to recent studies, various drugs induce a low level of PPARγ activation. These drugs may have the simultaneous functions of neuroprotection and fat metabolism and may weaken the side effects of drugs activating PPARγ. In the future, the partial agonist of PPARγ (such as telmisartan, mesalazine, *etc*.) might be added to the treatment of clinical diabetes as a protective agent to reduce the risk of neurological complications in patients with diabetes.

In conclusion, a PPARγ activator inhibits oxidative stress and reduces ferroptosis through the synergistic effect of the PPARγ and Nrf2 pathways. The PPARγ and Nrf2 pathways also can regulate iron metabolism, which may be another molecular mechanism that inhibits ferroptosis. The function of PPARγ agonists in inhibiting ferroptosis may become a new therapeutic target in the treatment of neurodegenerative diseases and tumors. More preclinical and clinical studies are needed to explore these possibilities further.

## Data Availability

The original contributions presented in the study are included in the article/Supplementary Material, further inquiries can be directed to the corresponding author.
