# Supplementary figures and images for: Activation of the PPARγ Prevents Ferroptosis-Induced Neuronal Loss in Response to Intracerebral Hemorrhage Through Synergistic Actions With the Nrf2
Source: Front Pharmacol. 2022 Apr 20;13:869300. doi: 10.3389/fphar.2022.869300 (PMC9065416; doi:10.3389/fphar.2022.869300)

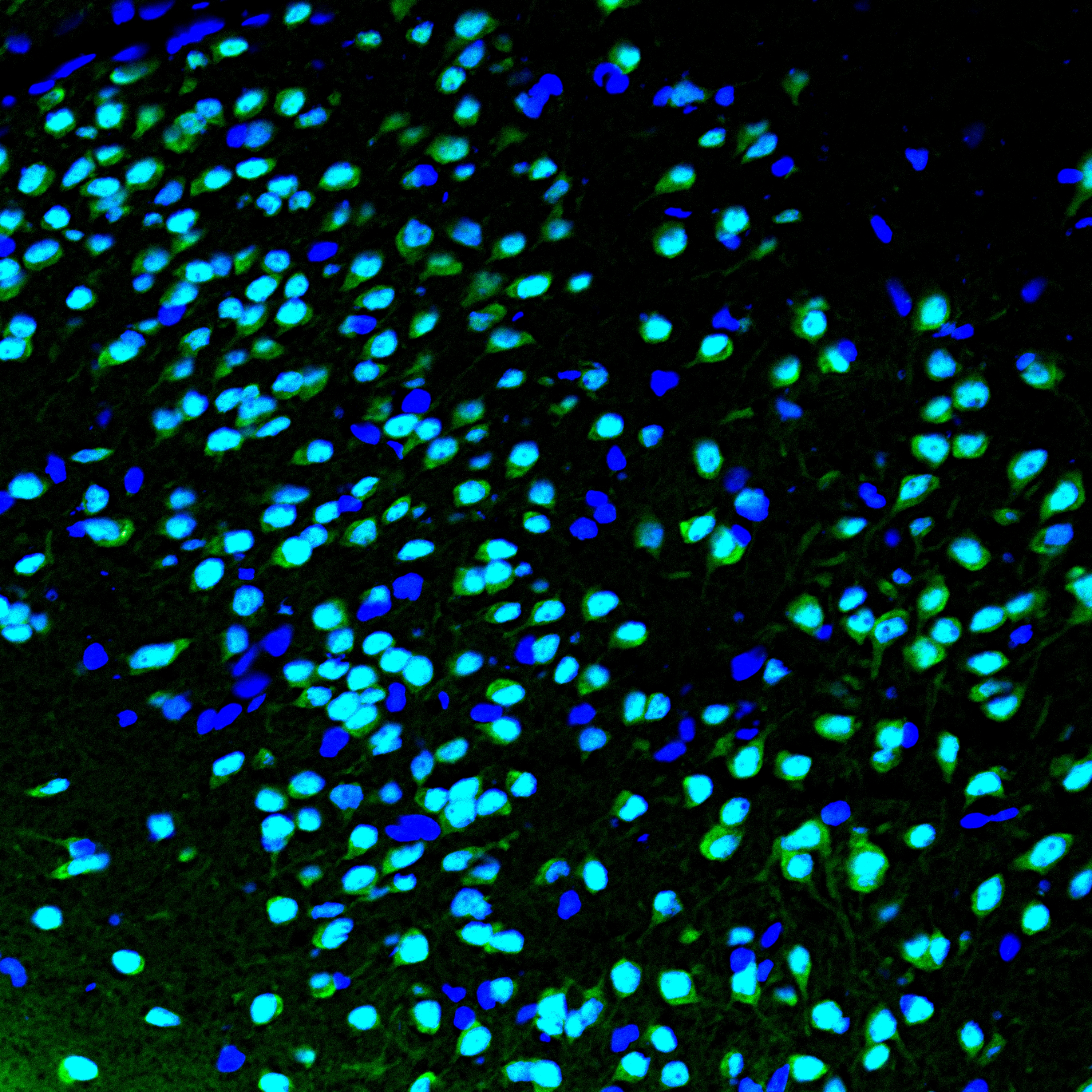

Supplement: Supplementary file 2 [file Image3.JPEG]

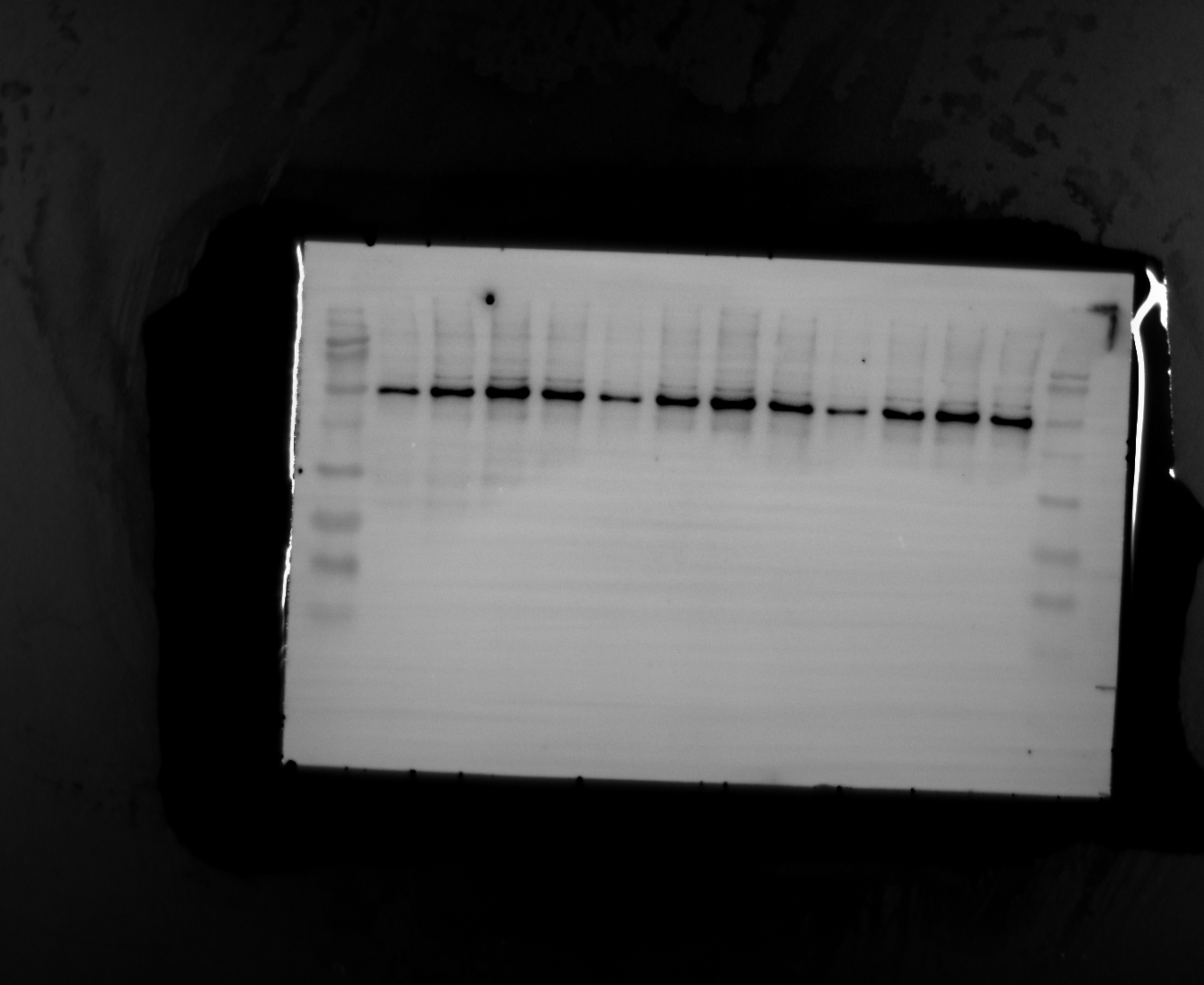

Supplement: Supplementary file 4 [file Image6.TIF]

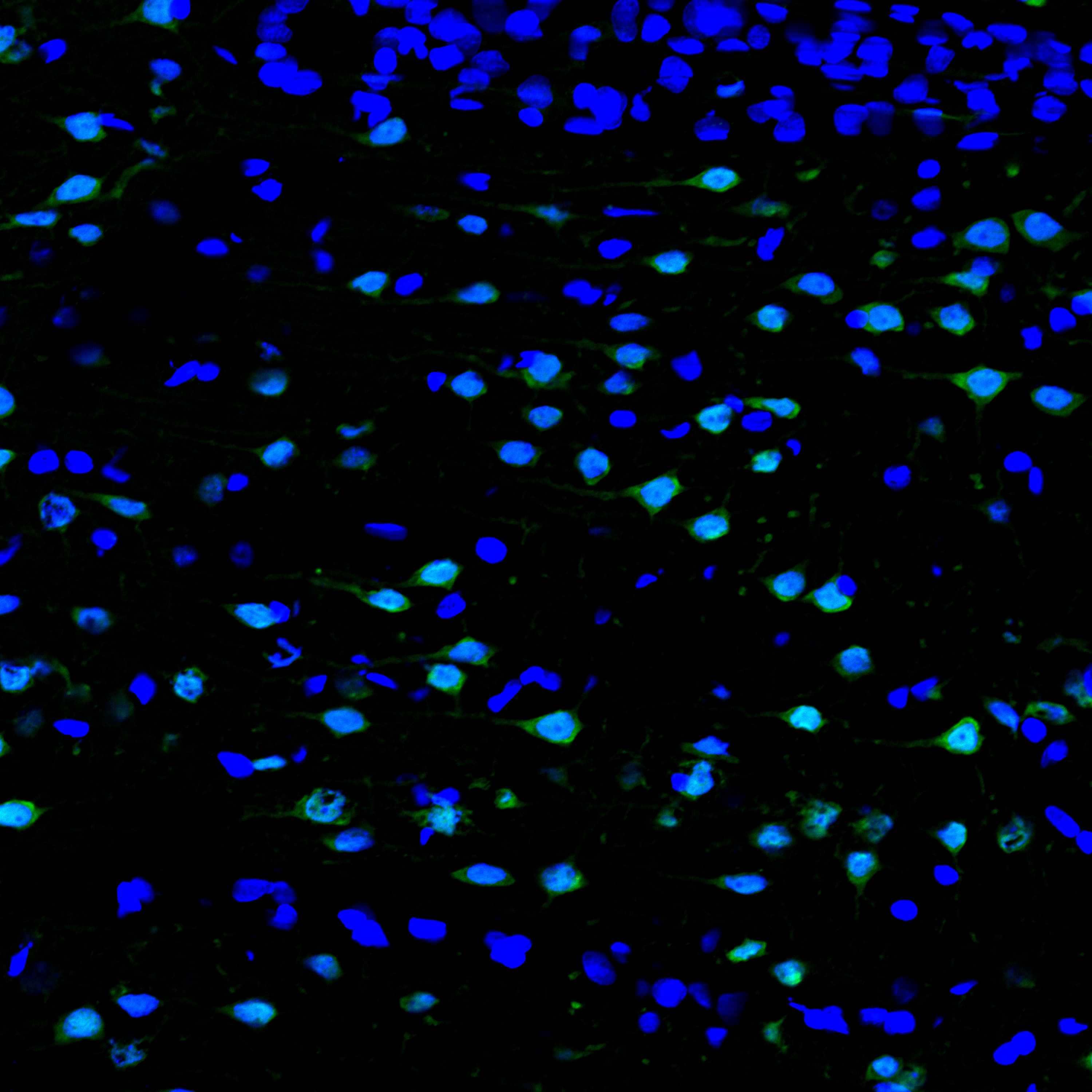

Supplement: Supplementary file 6 [file Image1.JPEG]

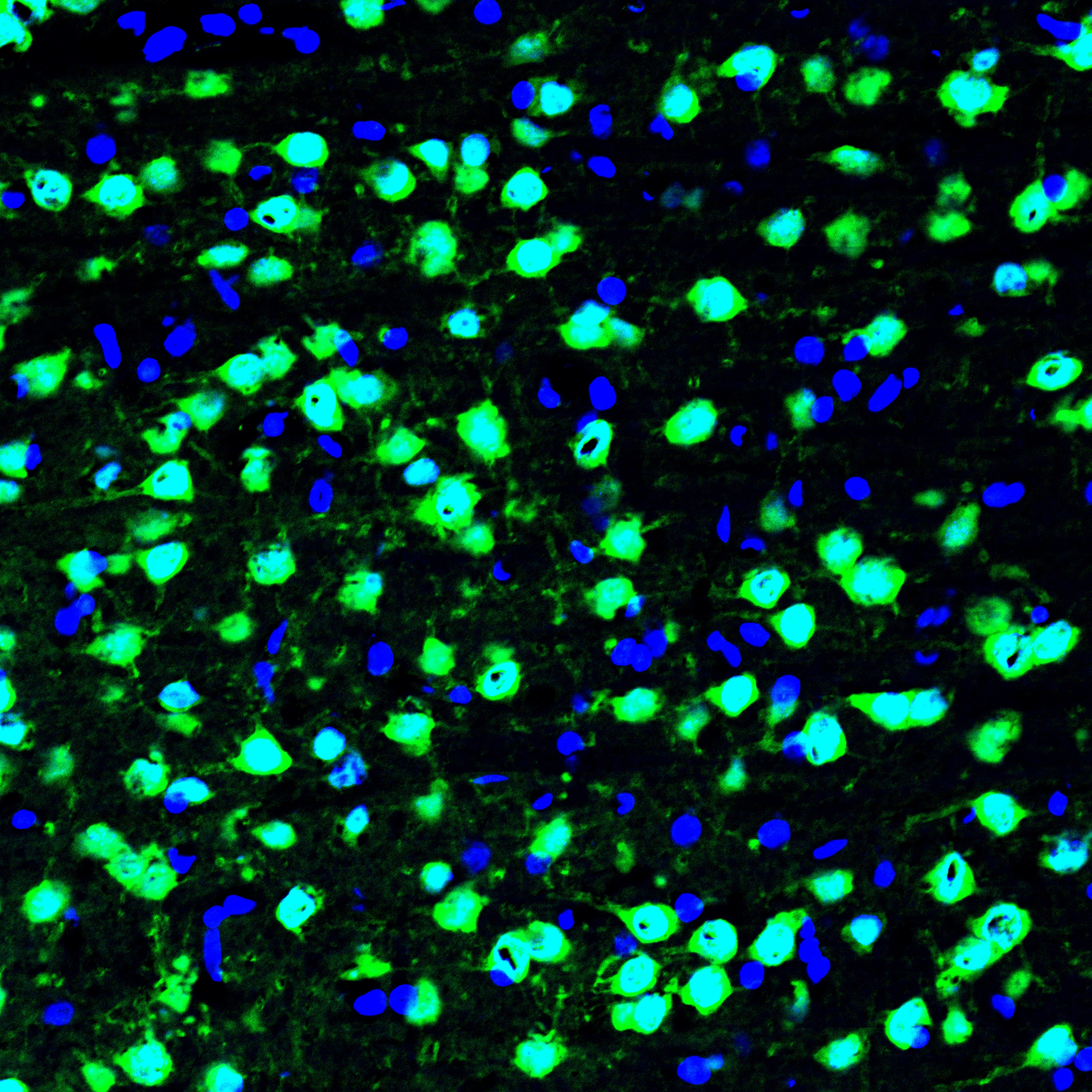

Supplement: Supplementary file 7 [file Image4.JPEG]

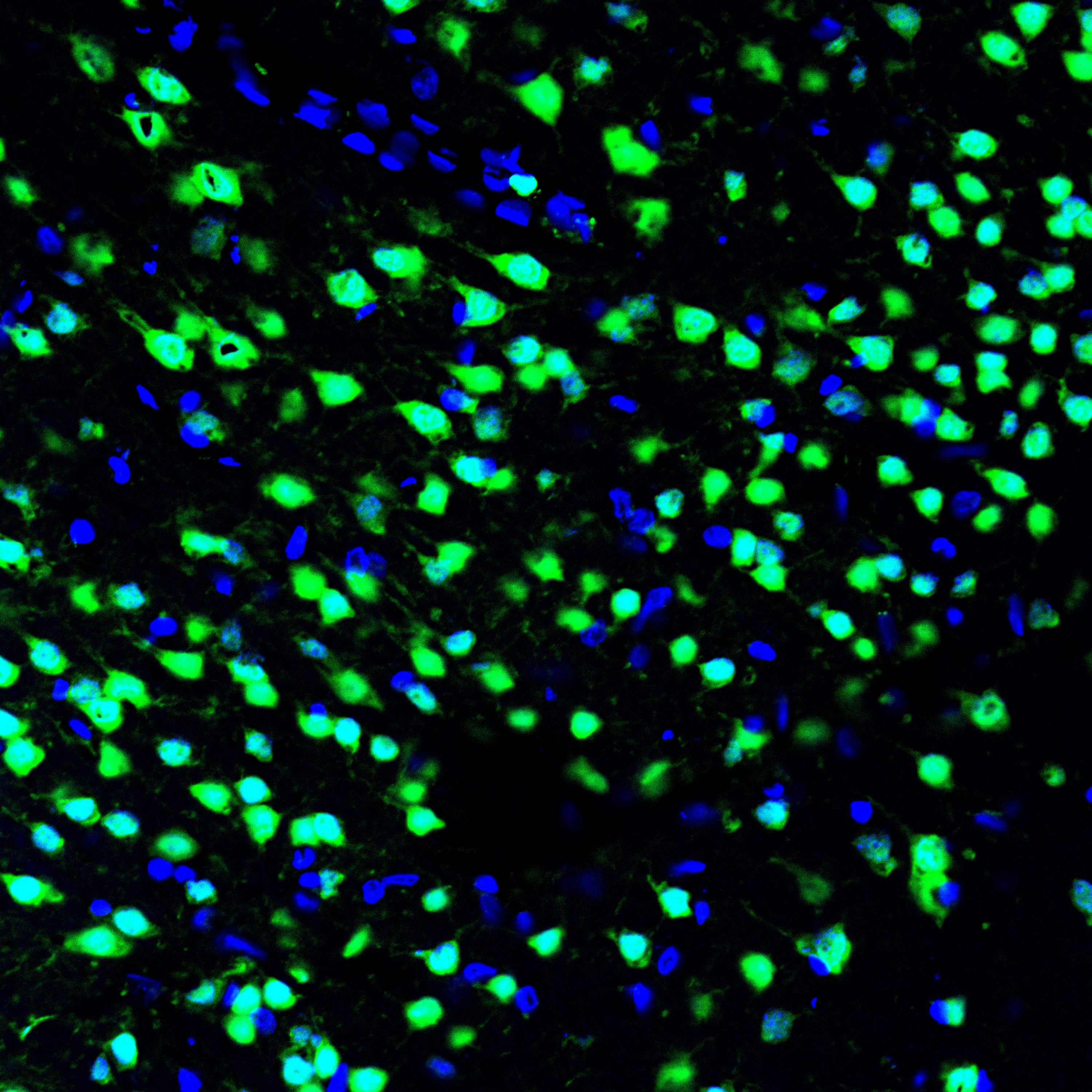

Supplement: Supplementary file 8 [file Image2.JPEG]

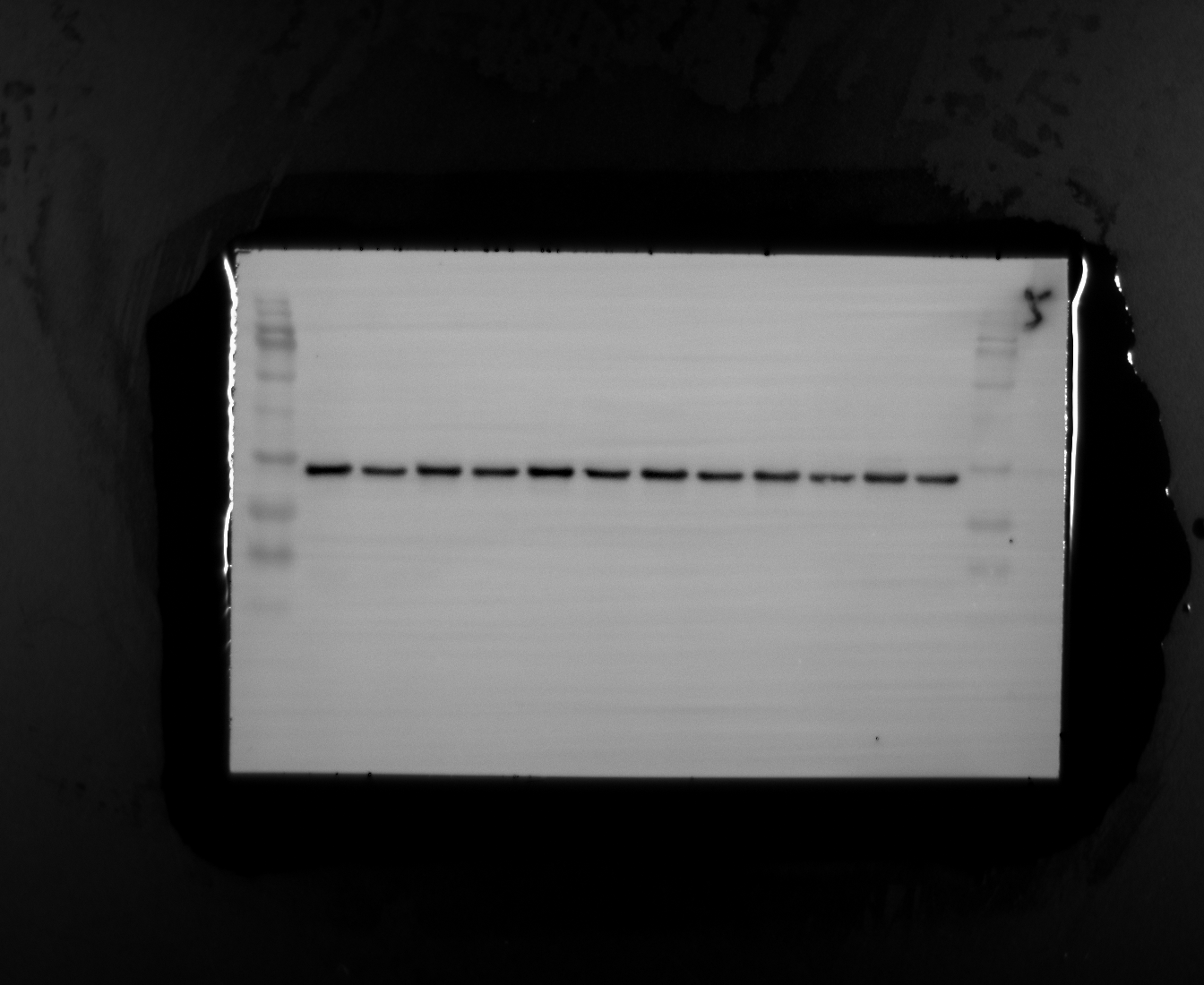

Supplement: Supplementary file 9 [file Image7.TIF]

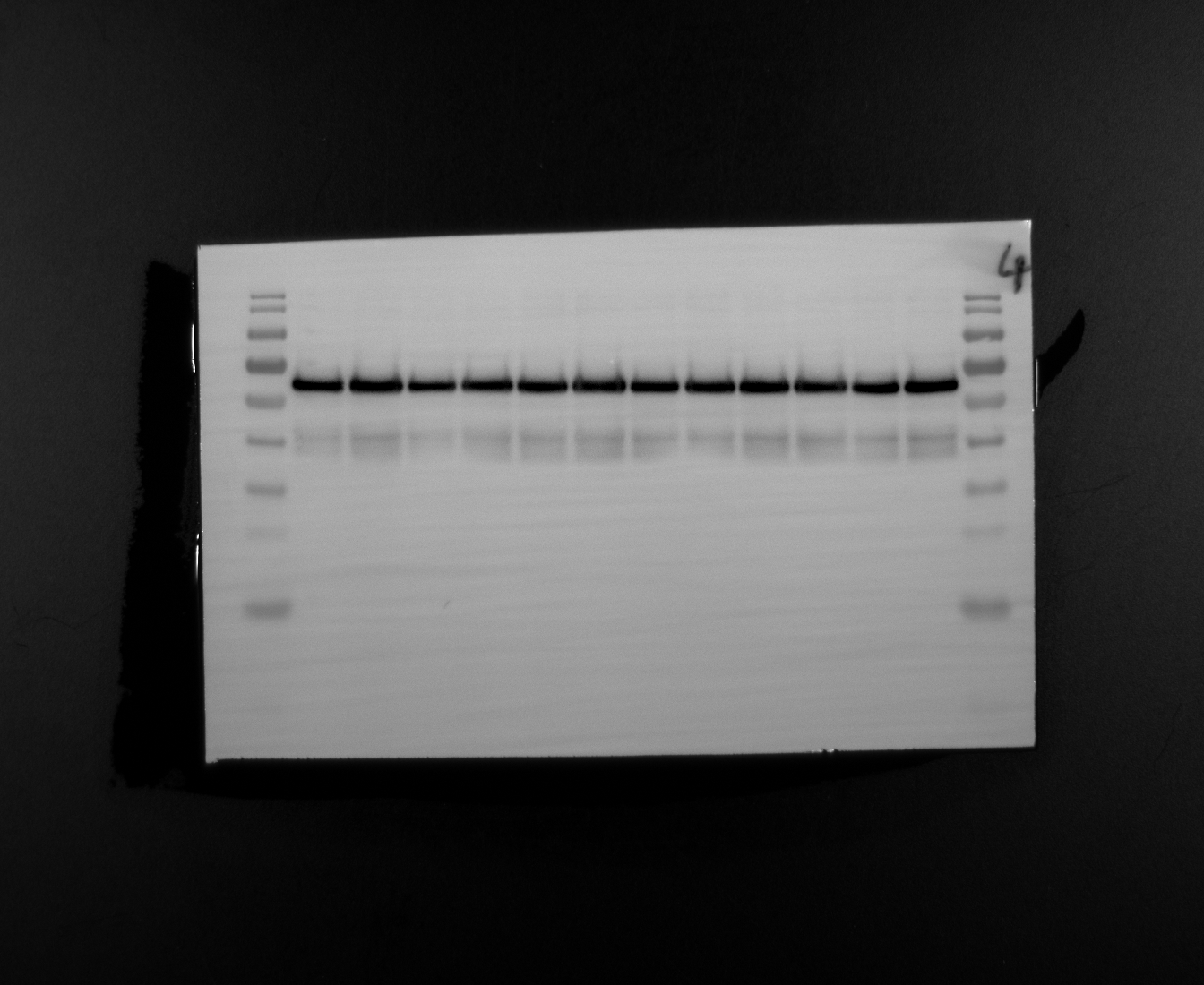

Supplement: Supplementary file 13 [file Image8.TIF]

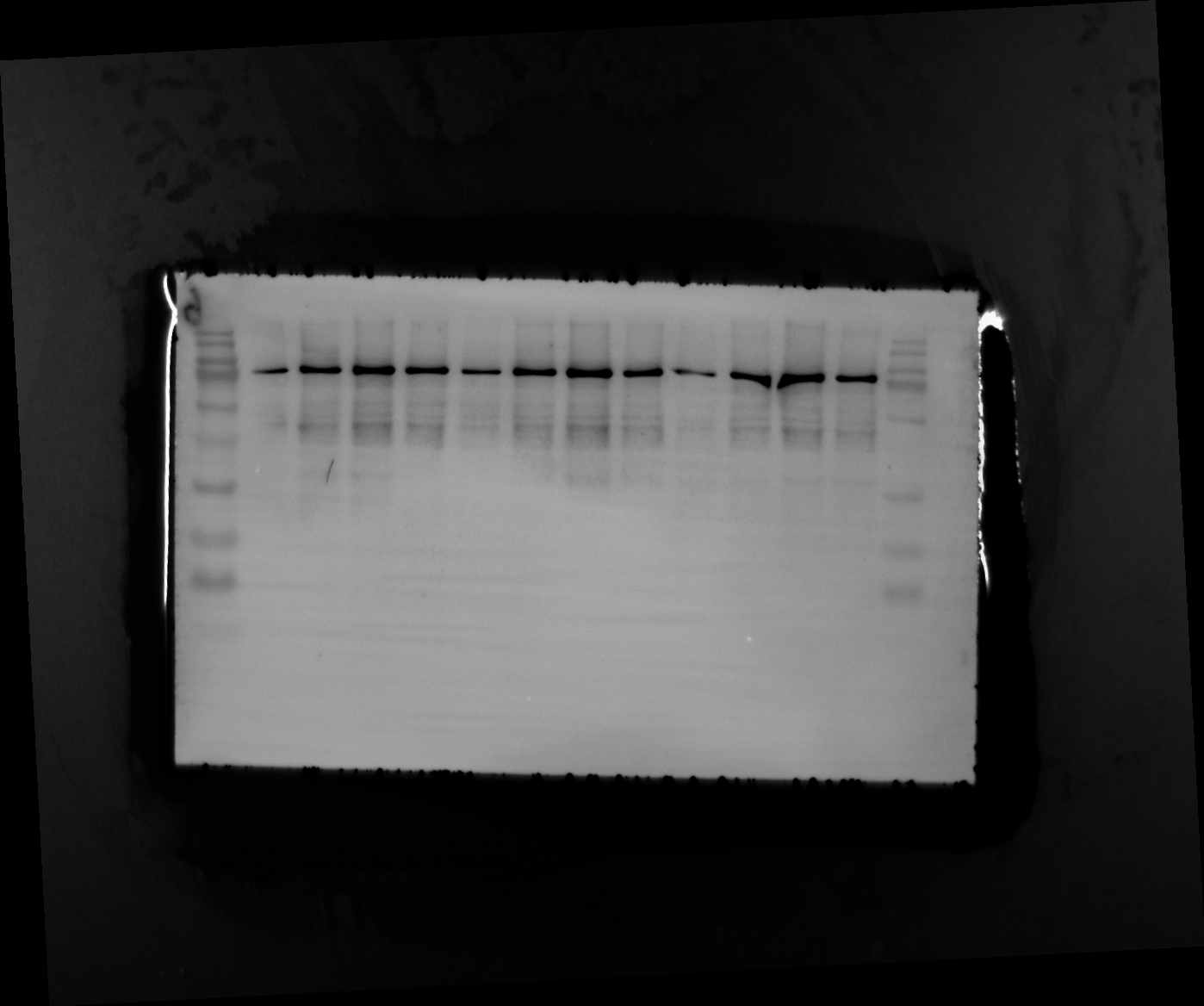

Supplement: Supplementary file 14 [file Image5.TIF]
